# First-Year College Students' Mental Health in the Post-COVID-19 Era in Guangxi, China: A Study Demands-Resources Model Perspective

**DOI:** 10.3389/fpubh.2022.906788

**Published:** 2022-06-13

**Authors:** Changwu Wei, Yan Ma, Jian-Hong Ye, Liying Nong

**Affiliations:** ^1^College of Education and Music, Hezhou University, Hezhou, China; ^2^Dhurakij Pundit University, Bangkok, Thailand; ^3^School of Foreign Studies, Hezhou University, Hezhou, China; ^4^Faculty of Education, Beijing Normal University, Beijing, China

**Keywords:** time pressure, perceived social support, emotional exhaustion, student engagement, mental health, first-year college students

## Abstract

The post-COVID-19 era means that the COVID-19 is basically under control; however, the risk of the pandemic still affects people's work, study, and life, physically and psychologically. In this era, due to the more challenges first-year college students face, more attention should be paid to their mental health. An emerging study demands-resources (SD-R) model can explain the influencing mechanism of college students' mental health. This model suggests that study demands increase the risk of student burnout, which results in mental health problems; meanwhile, study resources reduce student burnout and increase student engagement, thus improving mental health. Based on the SD-R model, this study explores the impacts of time pressure, emotional exhaustion, perceived social support, and student engagement on mental health and provides adequate measures to reduce the risk of mental health problems among first-year students. Time pressure, perceived social support, emotional exhaustion, student engagement, and mental health scales were used to investigate 537 first-year students at three universities in Guangxi, China, of whom 290 (54%) were female, and 247 (46%) were male, and the average age was 18.97 ± 1.01. Results indicated that: (1) Moderate scores on time pressure and emotional exhaustion and slightly-above-the-median scores on perceived social support, student engagement, and mental health were found among first-year students in the post-COVID-19 era. (2) Time pressure had a positive relationship with emotional exhaustion and a negative relationship with mental health. (3) Perceived social support was negatively correlated with emotional exhaustion but positively correlated with student engagement, and thus improved mental health. Results of this study with a sample of first-year college students in China support the hypotheses based on the SD-R model. These findings suggest that increasing perceived social support and student engagement while decreasing time pressure and emotional exhaustion may promote mental health among first-year college students.

## Introduction

Since 19 April 2020, China has been in a post- COVID-19 era. The post- COVID-19 era means that the COVID-19 is basically under control; however, the risk of the pandemic still affects people's work, study, and life, physically and psychologically. COVID-19 is a crucial public health issue that may lead to considerable mental health problems (1), and COVID-19-related factors are associated with long-term mental health symptoms among the general public (2). Moreover, studies on college students' mental health have been mounting steadily during and since COVID-19. For example, it was revealed that college students were at high risk of developing mental health issues during the pandemic (3), and 6 months after the COVID-19 outbreak, the risk of mental health problems and suicidal thoughts among students was relatively high (4).

Studies show that first-year college students are expected to manage their study time on their own (5), build new relationships, and change their learning styles (6). They experience interpersonal difficulties (7), academic pressure (8, 9), and other practical problems. As a result, they may face more challenges and suffer more mental health issues (6). Dropping out often occurs in the first year (10) and first-year college students are more likely to suffer from academic burnout and psychological problems than other college students (11). In addition, many students start university with low mental health levels (12), and some mentally healthy students may slip into a crisis of significant mental health problems during their first academic year (13). More than one-third of first-year students reported mental health problems (14), which means that more attention should be focused on the mental health of first-year students [e.g., (12, 13)].

However, first-year college students with positive personal qualities, such as self-efficacy and resilience, can navigate college, showing higher academic achievement and fewer mental health problems (9, 15, 16). Those with good social support (8, 17) or support from family and peers (18) have better academic performance, and perceived social support is beneficial to students' mental health (19). Based on the prior studies, this study attempted to explore the mechanism of negative factors (such as time pressure and emotional exhaustion) and positive factors (such as perceived social support and student engagement) on mental health.

An emerging study model, the study demands-resources (SD-R) model, discusses the influencing mechanism from both positive and negative processes. Study demands increase the risk of student burnout, leading to adverse outcomes like mental health problems. In contrast, study resources alleviate student burnout and benefit mental health by improving student engagement (20). This study was intended to apply the SD-R model to explore the influencing mechanism of mental health among first-year college students in the post-COVID-19 era. It adopted a sample of first-year college students who entered college in September 2021 and had 2 months of college life in Guangxi, China. The aim is to explore the mechanism of factors, such as time pressure, emotional exhaustion, perceived social support, and student engagement on mental health, and provide adequate measures to reduce the risk of mental health problems among first-year students.

## Literature Review

### Study Demands-Resources Model and Current Research Framework

The study demands-resources (SD-R) model was proposed by Lesener et al. (20). Two causal and independent processes are combined in this model. One is the health impairment process; the increased risk of student burnout caused by study demands results in adverse outcomes such as mental health problems. The other is the motivational process; study resources alleviate student burnout and benefit mental health by improving student engagement. This study intended to apply the SD-R model to explore the influencing mechanism on mental health.

Lesener et al. (20) defined *study demands* as physical, psychological aspects that require sustainable physical or mental effort, such as development challenges, time pressure, and interpersonal problems. *Student burnout* means students are feeling exhausted and incompetent due to study demands, becoming cynical or detached in manner toward one's studies, and experiencing emotional exhaustion (21). *Study resources* are valuable parts of studying that promote positive outcomes, such as mental health and academic success. Those valuable parts may be psychological, physical, organizational, or social (20), such as perceived social support and self-efficacy. *Student engagement* refers to a positive and satisfactory state of mind, such as vigor, dedication, and absorption (21).

Lesener et al. (20) proposed a broad range of study demands and resources for future studies. It has been demonstrated that the explanatory power of time pressure for study demands is greater than other variables, such as challenging, physiological, and psychological demands (22–24). Therefore, this study also adopted time pressure as an indicator of study demands to test the relations between variables in the SD-R model. Similarly, emotional exhaustion is a crucial indicator with the most significant explanatory power for student burnout (25). In addition, perceived social support is one of the study resources which has been most widely studied (20, 26–28). Therefore, variables in this study included time pressure, emotional exhaustion, perceived social support, student engagement, and mental health.

*Time pressure* refers to stress caused by academic burdens and short-term emergencies, such as lack of leisure time, too many obligations or responsibilities, heavy demands from extracurricular activities, and lack of enough time for sleep (29). *Perceived social support* is the perception of support from family, a teacher, and a friend (30). *Emotional exhaustion* is a long-term state of psychosomatic depletion due to constant stress from excessive work and personal demands (31). In this study, the student's *emotional exhaustion* is a state of physical and mental exhaustion due to study demands, such as time pressure and academic burdens. *Student engagement* refers to a positive and satisfactory state of mind described as vigor, dedication, and absorption (21). *Mental health* refers to psychological well-being and distress in the general population (32). Psychological well-being is a positive mental health state (such as happy, calm, and peaceful), while psychological distress is a negative state (such as anxiety and depression).

**Figure 1:**
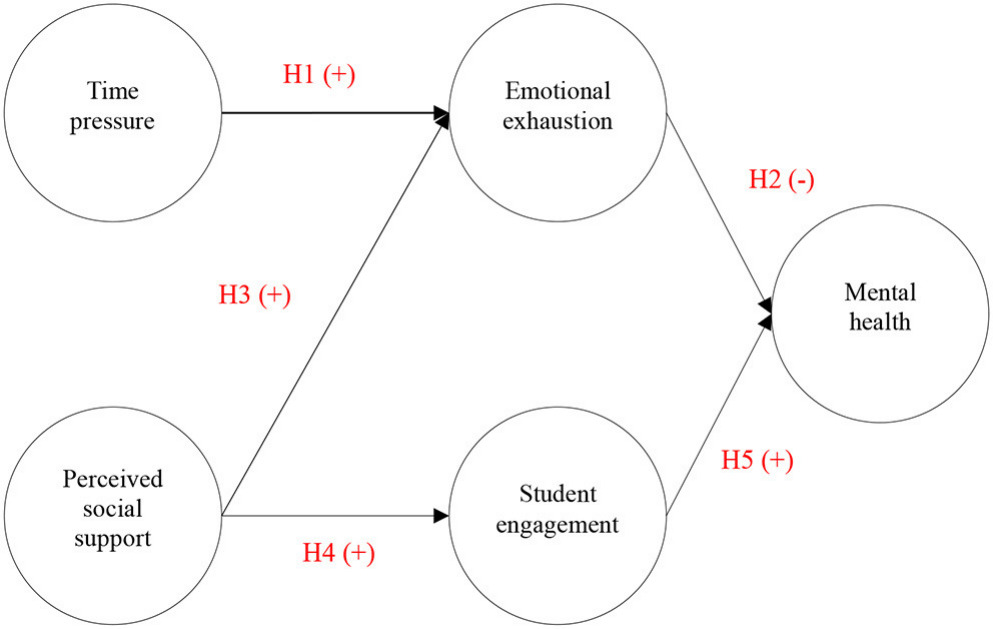
Research model.

Based on the SD-R model, this study was also intended to explore the influencing mechanism of mental health among first-year college students in the post-COVID-19 era through two processes. On the one hand, time pressure is positively related to emotional exhaustion, which impairs mental health; on the other hand, perceived social support is negatively related to emotional exhaustion and positively related to student engagement, which benefits mental health. The research framework of this study is shown in Figure 1.

### Time Pressure and Emotional Exhaustion

The explanatory power of time pressure for study demands is greater than other variables, such as challenging, physiological, and psychological demands (22–24). Emotional exhaustion is a crucial indicator with the most significant explanatory power for student burnout (25). Most studies indicate that time pressure positively relates to student emotional exhaustion (33, 34). That is to say, students who encounter high time pressure may experience high levels of emotional exhaustion. For those students with part-time jobs, time pressure was positively related to emotional exhaustion (35). In addition, study demands have a longitudinal effect on school burnout a year later (36). Therefore, based on the studies above, hypothesis 1 was proposed as follows:

H1: Time pressure relates to emotional exhaustion positively.

### Emotional Exhaustion and Mental Health

Studies show that emotional exhaustion is a negative indicator of mental health (37, 38). Greater emotional exhaustion is associated with poorer mental health (39). Students with higher levels of emotional exhaustion have more mental health problems (40, 41). Studies also found that emotional exhaustion harms mental health during the COVID-19 pandemic (42). Therefore, in line with the findings in prior studies, hypothesis 2 was proposed as follows:

H2: Emotional exhaustion negatively relates to mental health.

### Perceived Social Support and Emotional Exhaustion

Studies show that perceived social support is significantly related to emotional exhaustion (43, 44). Social support from school or teachers, parents, and peers was negatively correlated with emotional exhaustion (27, 45–47). Meanwhile, social support helps reduce study burnout, including emotional exhaustion like dejection (48). Therefore, in line with these research findings, hypothesis 3 was proposed as follows:

H3: Perceived social support negatively relates to emotional exhaustion.

### Perceived Social Support and Student Engagement

Studies indicate that perceived social support positively predicts student engagement (43, 49). The teacher-student relationship is an essential factor affecting student engagement (50–52). Support from teachers, peers, and family members positively impacts student engagement (53–55). Therefore, in line with the findings in the prior studies, hypothesis 4 was proposed as follows:

H4: Perceived social support positively relates to student engagement.

### Student Engagement and Mental Health

Studies suggest that student engagement is positively correlated with mental health (56, 57). Students with higher engagement have better mental health (58, 59). Recently, some scholars pointed out that student engagement partially mediates perceived social support and mental health (49). Therefore, in line with these findings, hypothesis 5 was proposed as follows:

H5: Student engagement positively relates to mental health.

Student mental health is an essential issue in higher education. More and more students have to be confronted with mental health issues. The more challenges first-year college students face, the more attention should be paid to their mental health. This study took first-year college students as research subjects to explore the factors affecting their mental health. Thus, the findings may contribute to the study on first-year college students' mental health characteristics in China and help provide some practical suggestions to alleviate the risk of mental health issues.

Moreover, time pressure may impair mental health by increasing student emotional exhaustion. Simultaneously, perceived social support may benefit mental health by decreasing emotional exhaustion and increasing student engagement. Thus, the findings may enrich the empirical studies of the SD-R model and offer empirical evidence for promoting mental health.

## Research Design

### Procedures

Adopting convenience sampling, this study was to investigate the relationship between time pressure, perceived social support, emotional exhaustion, student engagement, and mental health relating to first-year college students. The anonymous cross-sectional survey was conducted online from November 1–30, 2021 (nearly 2 months after entering college). First-year college students from nine classes at three universities (three classes per university) in Guangxi, China participated in this survey. Three mental health education teachers (one from per university) instructed the students to complete the questionnaire with informed consent in the introduction section.

#### Participants

Five hundred and eighty-eight questionnaires were received, 51 invalid questionnaires were excluded, and 537 valid samples (91.3%) were left for statistical analysis. Among them, 290 (54%) were females, and 247 (46%) were males; the average age of the respondents was 18.97 ± 1.01 years; 364 students (67.8%) were liberal arts majors, and 173 students (32.2%) were science majors, as shown in Table 1.

**Table 1:** Description of demographics.

	**Number or M ±SD**	**Percentage**
**Gender**
Male	247	46%
Female	290	54%
**Age**	18.97 ± 1.01	
**Major**
Liberal arts	364	67.8%
Science	173	32.8%

### Questionnaire

The questionnaire contained scales measuring time pressure, perceived social support, emotional exhaustion, student engagement, and mental health. The items in the questionnaire were developed from relevant literature.

Three educational management experts and a doctor of psychology examined the accuracy and applicability of item translation to ensure content validity, and 10 students participated in a test to ensure face validity. The Likert scale employed in each scale in this study was the same as the original scales (see the introductions of the measurement tools below). The reliability and validity of items in the questionnaire were listed in Tables 2, 3.

**Table 2:** Results of CFA Fit Indexes and Discriminant Validity of items.

**Index**	**χ2/*df***	**RMSEA**	**GFI**	**AGFI**	***t***
Threshold	<5	<0.10	>0.80	>0.80	>3
Time pressure	3.01	0.06	0.99	0.97	6.09–14.46
Perceived social support	2.79	0.07	0.96	0.93	4.77–12.39
Emotional Exhaustion	1.69	0.04	0.99	0.98	36.62–41.52
Student engagement	4.46	0.07	0.97	0.95	21.93–31.37
Mental health	3.16	0.06	0.99	0.97	17.43–24.84

**Table 3:** Reliability, Validity, Factor Loading (FL), and Scores of Each variable.

**Variables**	**α**	**CR**	**AVE**	**FL**	**M ±SD**	**Maximum**
Criteria	>0.70	>0.70	>0.50	>0.50	–	–
Time pressure	0.75	0.84	0.51	0.60–0.76	2.57 ± 0.60	4
Perceived social support	0.96	0.97	0.79	0.86–0.92	3.84 ± 1.01	5
Emotional Exhaustion	0.92	0.92	0.75	0.79–0.92	3.86 ± 1.47	7
Student engagement	0.92	0.92	0.65	0.70–0.90	3.25 ± 0.86	5
Mental health	0.80	0.81	0.52	0.50–0.85	2.80 ± 0.57	4

#### Time Pressure

This study employed a seven-item time pressure scale to measure students' study-related time pressure. It is a subscale of the Inventory of College Students' Recent Life Experiences (ICSRLE) (29). A sample item is “I always have many learning tasks to deal with.” Two items were deleted because the factor loadings were lower than 0.50. The deleted items were “Not enough time for sleep” and “A lot of responsibilities.” A four-point Likert scale was used (1 = *never* to 4 = *always*).

#### Perceived Social Support

Zimet et al. (60) developed the Multidimensional Scale of Perceived Social Support (MSPSS), which comprises 12 statements indicating three dimensions of perceived support (family, friends, and significant others), with four items for each dimension. The Chinese version of MSPSS, revised by Jiang (61), was used to measure students' perceived support from family members, peers, and teachers, with “significant others” replaced by “teachers” in this study. Items, for example, were “I can share my problems with friends” (perceived support from friends), “My family tries to help me” (perceived support from family), or “My teacher always offers emotional support to me” (perceived support from teachers). A five-point Likert scale was used (1 = *strongly disagree* to 5 = *strongly agree*).

#### Emotional Exhaustion

Ni et al. (62) revised the emotional exhaustion subscale from the Maslach Burnout Inventory—General Survey (MBI—GS) (63). The revised version comprises four items. In this study, the four-item subscale was employed to measure emotional exhaustion related to study among the first-year college students in Guangxi, China. A sample item is: “I feel exhausted at the end of a day at university.” A seven-point Likert scale was used (1 = *never* to 7 = *always*).

#### Student Engagement

The nine-item Utrecht Work Engagement Scale-Student Form (UWES-9-SF) (21) consists of three subscales, including vigor, dedication, and absorption, with three items for each. The UWES-9-SF was used to measure student engagement among first-year college students. A sample item is as follows: “I feel happy when studying intensively.” It was found in this study that the USES-9-SF is a unidimensional scale. Three items were deleted because the factor loadings were lower than 0.50. The deleted items were as follows: “At my study, I feel bursting with energy,” “When I am learning, I feel that time flies,” and “When I am learning, I forget everything else around me.” A five-point Likert scale was used (1 = *strongly disagree* to 5 = *strongly agree*).

#### Mental Health

This study employed the Mental Health Inventory 5 (MHI-5) to measure the mental health of first-year college students. It was compiled by Berwick et al. (64), containing five items evaluating how long the following states were felt in the last month: Happy, calm, nervous, depressed, and so depressed that I cannot pull myself together. A sample item is: “During the last month, how much of the time have you been a very nervous person?” A four-point Likert scale was used (1 = never to 4 = always), assessing two positive items and three negative items on mental health, and the three negative items were scored in reverse.

## Results

### Scores on the Study Variables

Scores for time pressure, emotional exhaustion, perceived social support, student engagement, and mental health are recorded in Table 3. Moderate levels of time pressure (*M* = 2.57, *SD* = 0.60, maximum was 4) and emotional exhaustion (*M* = 3.86, *SD* = 1.47, maximum was 7) were found, and slightly-above-the-median levels of perceived social support (*M* = 3.84, *SD* = 1.01, maximum was 5), student engagement (*M* = 3.25, *SD* = 0.86, maximum was 5), and mental health (*M* = 2.80, *SD* = 0.57, maximum was 4) were found among the first-year students.

### Measurement Model

In this study, SPSS 26.0 was used to calculate the Cronbach's α of each scale. Cronbach's α value of each component was between 0.75 and 0.96, indicating acceptable reliability. AMOS 23.0 was used for confirmatory factor analysis to examine the factor loadings, composite reliability (CR), and average variance extracted (AVE). This study employed the criteria of fit indexes in CFA suggested by Hair et al. (65). As shown in Table 2, all the χ^2^/*df* values were <5, GFI and AGFI were higher than 0.80, and RMSEA were <0.10, indicating an acceptable model.

#### Convergent Validity

According to the convergence validity evaluation criteria (66), the acceptable factor loading value should be at least 0.50; composite reliability (CR) should be at least 0.7; and the average variance extracted (AVE) value should be at least 0.50. As shown in Table 3, the factor loading values of each dimension were between 0.77 and 0.87; The CR values were between 0.81 and 0.97, and the AVE values of the dimensions were between 0.59 and 0.77, indicating an acceptable convergent validity for each dimension.

#### Discriminant Validity

The square root of the AVE should be greater than the correlation coefficient between the two dimensions (67). As shown in Table 4, the square root of the AVE for each dimension was between 0.77 and 0.86, which was greater than the correlation coefficient between the dimensions, representing an acceptable discriminant validity for each dimension.

**Table 4:** Discriminant validity analysis.

**Construct**	**1**	**2**	**3**	**4**	**5**
(1) Time pressure	**0.71**				
(2) Perceived social support	−0.60	**0.86**			
(3) Emotional Exhaustion	0.61	−0.67	**0.89**		
(4) Student engagement	−0.22	0.33	−0.32	**0.80**	
(5) Mental health	−0.34	0.44	−0.43	0.39	**0.72**

*Note: The bold value indicates the square root of the AVE for each dimension*.

### Structural Model

#### Model Fit Index

AMOS 23.0 statistical software was used to check the fitness of the research model. The model fit index standards recommended by Hair et al. (65) are RMSEA should be lower than 0.10, GFI, AGFI, NFI, and NNFI should be at least 0.90, CFI, IFI, and RFI should be at least 0.80, PNFI and PGFI should be at least 0.50. This study reported fit index values as follows: RMSEA = 0.07, GFI = 0.85, AGFI = 0.83, NFI = 0.90, NNFI = 0.92, CFI = 0.93, IFI = 0.93, RFI = 0.89, PNFI = 0.82, and PGFI = 0.72. Most of the fit indexes of the model in this study met the criteria, indicating it was an acceptable model.

#### Path Analysis

**Figure 2:**
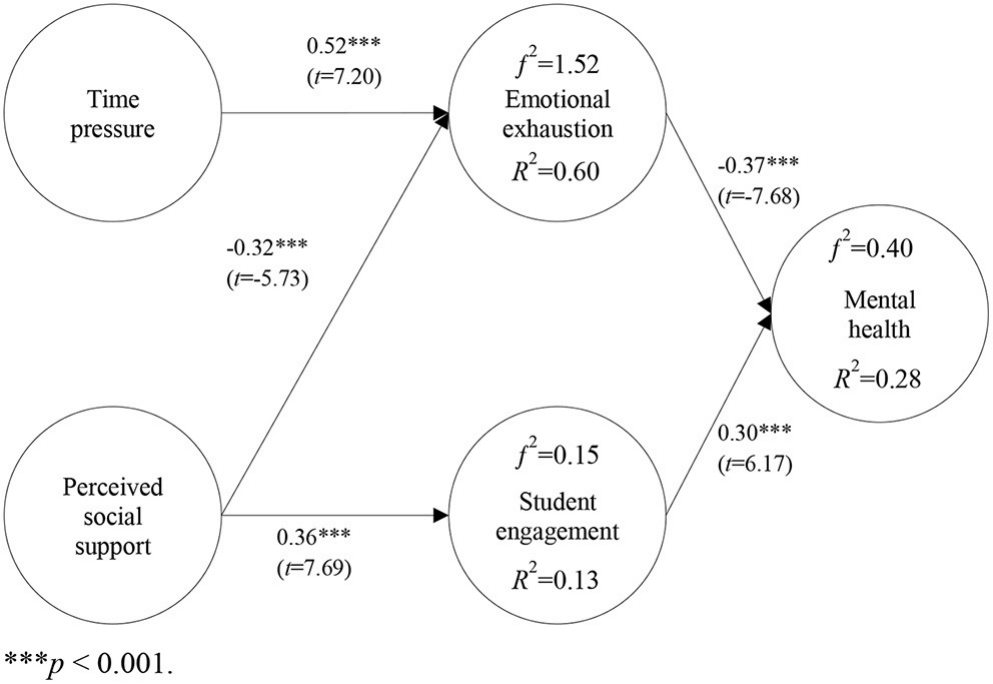
Verification of the Research Model. ***p < 0.001.

Data obtained through Path analysis can be seen in Figure 2. Findings revealed that the coefficient in each path was significant (*p* < 0.001). Time pressure had a positive association with emotional exhaustion (β = 0.52). Emotional exhaustion had a negative association with mental health (β = −0.37). Perceived social support had a negative association with emotional exhaustion (β = −0.32). Perceived social support had a positive association with student engagement (β = 0.36); student engagement had a positive association with mental health (β = 0.30).

The value of *R*^2^ represents the compounding effects of the exogenous latent variables on the endogenous ones. *R*^2^-values of 0.75, 0.50, or 0.25 indicate significant, moderate, or weak coefficients of determination, respectively (68). Time pressure and perceived social support accounted for 60% of emotional exhaustion; perceived social support accounted for 13% of student engagement. Emotional exhaustion and student engagement accounted for 28% of mental health.

Effect size *f*^2^ is calculated by the formula *f*^2^= *R*^2^/(1–*R*^2^). Effect sizes *f*^2^ of 0.02, 0.15, and 0.35 indicate small, medium, and significant effects of exogenous latent variables, respectively (69). Time pressure and perceived social support accounted for emotional exhaustion with an effect size *f*^2^ of 1.52, indicating significant effects of these two exogenous latent variables. Perceived social support accounted for student engagement with an effect size *f*^2^ of 0.15, indicating a medium effect of this exogenous latent variable. Student burnout and student engagement accounted for mental health with an effect size *f*^2^ of 0.40, indicating significant effects of these two exogenous latent variables.

## Discussion

### Scores on the Study Variables

Studies show that COVID-19 has a moderate but sustained impact on the mental health of first-year students (70). Van Zyl et al. (71) found in a sample of university students in the Netherlands that the scores on study demands are slightly lower than the median, and the scores on mental health and perceived support from peers and teachers are higher than the median. This study revealed that the time pressure scores of the first-year college students in Guangxi are consistent with those of the Dutch sample. Namely, it is also slightly lower than the median. The difference is that the mental health and perceived social support scores were close to the median. In addition, studies show that college students have opposite scores on student burnout and study engagement. For example, Barratt and Duran (43) found that students score high on student engagement but low on student burnout in a UK sample of an online teaching project. A study from Poland also shows that scores on student engagement are opposite to student burnout scores (72). However, this study revealed that both emotional exhaustion (student burnout) and student engagement scored near the median, and they did not score oppositely. The results of this study enrich the studies on characteristics of mental health of first-year college students in Guangxi in the post-COVID-19 era.

### Hypotheses Based on the Health Impairment Process

Lee and Ashforth (73) believed that compared with cynicism and inefficacy in job burnout, emotional exhaustion has a higher correlation with demands (including time pressure as an indicator) and resource variables. More recently, Kim et al. (33) found a positive correlation between study demands and emotional exhaustion in the school setting through a meta-analysis. Similar findings show a significant positive correlation between study demands and emotional exhaustion among high school students (34) and college students (20). This study also reported that the time pressure of first-year students in Guangxi colleges was positively correlated with emotional exhaustion, and therefore, hypothesis 1 of this study is supported.

Studies before the COVID-19 outbreak show that student burnout is negatively correlated with the mental health of college students. In other words, student burnout is positively related to mental health problems, including depression, anxiety (41), and psychopathological symptomatology (74). A COVID-19- related study on the correlation of emotional exhaustion with mental health revealed that, around the outbreak of COVID-19, the mental health of college students decreased with the increase of student burnout, especially emotional exhaustion (37, 75). A similar finding reported that student burnout is positively correlated with depression among medical students (76). This study also supports these findings; namely, the emotional exhaustion of first-year students in Guangxi colleges was negatively correlated with their mental health.

In the health impairment process in the SD-R model, time pressure, as an essential indicator of study demands, is positively related to emotional exhaustion (an indicator of student burnout), which is negatively correlated with mental health. As discussed above, the results of this study provide empirical evidence of the health impairment process of SD-R, just as Gusy et al. (22) did in a longitudinal study of SD-R's health impairment process.

### Hypotheses Based on the Motivation Process

Lee and Ashforth (73) claimed that resource variables are negatively related to emotional exhaustion. As one of the study resources, perceived social support has been widely discussed in a variety of studies (20, 26–28). Many studies show that social support is negatively correlated with student burnout (27, 33, 48). Social support from school or teachers, parents, and peers is negatively correlated with student burnout (27, 45–47); that is probably because social support has a promotive effect on reducing student emotional exhaustion (48). However, few studies have explored the relationship between social support and emotional exhaustion in the post-COVID-19 era. These studies mainly focus on occupational groups, such as medical professionals (77). The finding of the present study may fill this research gap. This study indicated that perceived social support was negatively related to emotional exhaustion among first-year college students in the post-COVID-19 era.

Many studies have focused on the correlation of perceived social support with job engagement in the context of COVID-19. For example, organizational support perceived by nurses during the COVID-19 pandemic was significantly correlated with job engagement (78, 79), and teachers' perceived organizational support had a positive impact on their job engagement (80). Similar results were also found in drivers (81), service workers (82), and technological workers (83). Studies have demonstrated that perceived social support positively relates to student engagement (43, 49); that is probably because social support plays a promotive role in increasing student engagement (53–55). However, few studies have explored the correlation of perceived social support with student engagement of college students in the context of COVID-19. Recent studies show that social support as a significant study resource positively impacts student engagement in colleges during the pandemic (84). The more support college students receive from their teachers, the higher their student engagement is (85). The finding of the present study may enrich studies on the correlation of perceived social support with student engagement of college students in the context of COVID-19.

Students with higher student engagement have better mental health (58, 59); that is to say, student engagement is positively correlated with mental health (56, 57). However, few studies explore the relationship between student engagement and mental health in terms of the environment in which students engage in their studies and cope with their psychological problems. This study was conducted under the background of COVID-19, adopting a sample of first-year students and exploring the factors which may influence their mental health. The results showed that student engagement of first-year students in Guangxi colleges after the pandemic was significantly correlated with their mental health.

In line with the motivation process of the SD-R model, the findings in this study revealed that perceived social support (study resources) was positively related to student engagement but negatively related to emotional exhaustion, and student engagement was positively related to mental health. These findings supported the motivation process of the SD-R model, just as the previous studies have shown (71, 86).

Based on the discussion above, the results in the current study offered empirical evidence to support the SD-R model in first-year college students in Guangxi, China, which may contribute to the application of the SD-R model in first-year college students, especially in the post-COVID-19 era.

In addition, this study revealed the impacts of time pressure, perceived social support, emotional exhaustion, and student engagement on mental health. The findings may contribute to the study on the characteristics of mental health among the first-year college students in China in the post-COVID-19 era and help explore some practical ways to alleviate the risk of mental health issues among them.

## Conclusions and Recommendations

### Conclusions

In terms of scores on time pressure, perceived social support, and mental health, a medium level was found in this sample and samples of other college students; namely, the findings in this study are consistent with those of the prior studies. However, scores on emotional exhaustion and student engagement were close to the median in this sample. In contrast, opposite scores were found in other samples in the previous studies. This study provides new findings on the mental health characteristics of first-year college students through a sample study of first-year college students in Guangxi in the post-COVID-19 era.

Results in this study offer empirical support for the SD-R model; that is, all the SD-R model hypotheses were supported. Time pressure (study demands) had positive effects on emotional exhaustion (student burnout), which negatively affected mental health. This finding supports the health impairment process of the SD-R Model. In addition, perceived social support (study resources) increased student engagement and decreased the emotional exhaustion of first-year students, thus promoting their mental health, which supports the motivational process of the SD-R Model.

From the above findings revealed in this study, we conclude that time pressure and perceived social support are critical factors that influence the mental health of first-year college students in the post-COVID-19 era. Both indicators impact the mental health of first-year students, the former by increasing emotional exhaustion, the latter by increasing student engagement and decreasing emotional exhaustion.

### Recommendations

Moderate levels of time pressure, emotional exhaustion, and a slightly above-average level of perceived social support, student engagement, and mental health were found among first-year students in the current study. We suggest that more attention should be paid to those students with low scores in mental health, which may indicate that they are suffering from mental health problems. Furthermore, we suggest that measures should be taken to reduce time pressure, which affects mental health by increasing emotional exhaustion. As for reducing time pressure, interventions should be adopted by modifying study programs or structural settings at universities (22); simultaneously, time management training may help reduce time pressure for university students (87). In addition, support from teachers, good campus environment perceptions, excellent class (socio-affective, design, and organization), and motivating teaching behavior are considered to help foster student engagement (88), thus, enhancing student mental health.

### Research Limitations and Future Study

First, one limitation of this study is that the sample was drawn from first-year college students in Guangxi, so the findings may not apply to all student groups. Future research should employ a stratified probability sample, investigating college students in different grades and regions.

Second, this cross-sectional study would not establish any causal relationship among the study variables. Longitudinal studies should be conducted in future research to examine the causal relationship among variables in the SD-R model.

Third, as one of the most crucial study demands, time pressure is a study demand that all students must face in most cases, but it is not representative enough to cover all indicators of study demands. Similarly, though perceived social support is a widely used study resource, it is unreasonable to employ it to stand for all study resources. That is to say, in future studies, other indicators concerning study demands (e.g., emotional demands, study load) and indicators related to study resources (e.g., self-efficacy, perceived classroom climate) should be taken into consideration.

## Data Availability Statement

The raw data supporting the conclusions of this article will be made available by the authors, without undue reservation.

## Ethics Statement

Ethical review and approval was not required for the study on human participants in accordance with the local legislation and institutional requirements. The participants provided their written informed consent to participate in this study.

## Author Contributions

CW, YM, and J-HY: concept and design, drafting of the manuscript, acquisition of data, and statistical analysis. CW, J-HY, and LN: critical revision of the manuscript. All authors contributed to the article and approved the submitted version.

## Conflict of Interest

The authors declare that the research was conducted in the absence of any commercial or financial relationships that could be construed as a potential conflict of interest.

## Publisher's Note

All claims expressed in this article are solely those of the authors and do not necessarily represent those of their affiliated organizations, or those of the publisher, the editors and the reviewers. Any product that may be evaluated in this article, or claim that may be made by its manufacturer, is not guaranteed or endorsed by the publisher.
